# A qualitative exploration of continuity of TB care in clinics after discharge from hospitals in Cape Town, South Africa

**DOI:** 10.1186/s12913-022-08880-9

**Published:** 2022-12-07

**Authors:** Idriss Ibrahim Kallon, Christopher J Colvin

**Affiliations:** 1 Division of Social and Behavioural Sciences, School of Public Health and Family Medicine, University of Cape Town, Cape Town, South Africa; 2 Centre for Evidence-based Health Care, Division of Epidemiology and Biostatistics, Department of Global Health, Stellenbosch University, Cape Town, South Africa; 3 Department of Public Health Sciences, University of Virginia, Virginia, USA; 4 Department of Epidemiology, Brown University, Providence, USA

**Keywords:** Tuberculosis, Continuity of care, Down-referred, Hospital, Clinic, South Africa

## Abstract

**Background:**

Continuity of care remains a challenge for TB patients who are discharged from hospital and referred to primary health care clinics in South Africa. The aim of this study was to explore the experiences and perceptions of patients, health care workers and family members regarding continuity of TB care in a Cape Town health district.

**Methods:**

We conducted one-on-one interviews, using semi-structured interview guides, with TB patients and their families and health care workers. We also conducted focus group interviews with other health care workers who performed similar duties. Field notes were kept and patients’ home circumstances were also physically observed. Data saturation was achieved after 31 interviews. We used Miles and Huberman’s qualitative data analysis framework to interpret the data.

**Results:**

Themes identified in the interviews were grouped into two categories: (1)  patients’ socio-economic circumstances including complex family relationships, good or lack of family support, inadequate income, and agency; and (2) health system challenges, including inadequate referral links between the clinic and the hospital and negative emotions as a result of poor service delivery experienced by patients.

**Conclusion:**

Some TB Patients experienced poor continuity of care on discharge from hospitals to primary health clinics and perceived that this resulted from socio-economic conditions and health system-related problems that triggered negative emotions. Proper communication between the hospital and clinic regarding patients’ care, adequate counselling, and patient-centred treatment are required to address poor continuity of care among patients with TB down-referred to clinics.

**Supplementary Information:**

The online version contains supplementary material available at 10.1186/s12913-022-08880-9.

## Introduction

By 2019, South Africa had an incidence of 360,000 active TB cases, which is a rate of about 615/100 000 [1]. About 14 000 contracted drug-resistant TB (DR-TB) the same year [1]. TB remains the leading cause of death in South Africa [2]. There are many drivers of TB incidence in South Africa, which include low coverage of antiretroviral therapy (ART) among TB patients co-infected with HIV [3] and low rates of TB screening and testing in the health system [3]. Amidst these problems, there is ineffective continuity of TB care in South Africa for patients discharged from hospitals who are expected to complete their treatment in primary health care (PHC) clinics [4–8].

Continuity of care in the South African context includes the proper transfer of patients’ information from one health facility to another, and/or ensuring adequate and consistent care for patients by different health workers over time during subsequent visits [4–6, 9]. The drivers of ineffective continuity of care include poor communication between the different tiers of healthcare facilities (for example, between the hospital and the clinic), disjointed monitoring and evaluation systems, those who have not been tested or treated for TB prior to discharge, and clinical hierarchies among the different cadres of staff that reduce effective communication and coordination [6, 10]. All these problems have been ongoing prior to, but exacerbated by, the COVID-19 pandemic as treatment of TB declined by 20% in 2020, compared to 2019 [11]. Qualitative studies have also identified problems including poor TB education and discharge planning prior to patient discharge from hospital [4, 7]. Most of these studies were conducted from health care workers’ (HCWs) perspectives and only a few from patients who have been diagnosed and initially treated with TB at the hospital [4, 6, 7, 12, 13]. The few studies that have drawn from patient perspectives have been examined the in-patient experience and discharge planning processes. In addition, few studies have evaluated continuity of care following patients discharged from hospital to PHC clinics [5, 7, 13]. The patients’ perspectives are key in understanding the problems of continuity of care because they are the recipients of care and have experienced TB treatment at the hospital, discharge processes in the hospital, and experience treatment at referral clinics. These experiences are also influenced by socio-economic challenges in their homes.

Some studies have identified the impact of socio-economic drivers of TB on continuity of care. These include family support (or lack of support), health-seeking behaviour, and low income [14–16]. In South Africa, although there is little evidence showing the relationship between low income, poverty and continuity of care, many TB patients are living in poor conditions with little or no income to sustain them in their treatment journey [15, 17]. These factors are likely to affect TB patients in South Africa in their pursuit for continuity of care in PHC. The aim of this study was to explore the experiences and perceptions of patients, health care workers and family members regarding continuity of TB care during down-referral in a Cape Town health district. This study focuses on both health system factors and the socio-economic influences of patients who have been diagnosed and initially treated with TB at the hospitals and who have continued their treatment at their referral clinics in South Africa.

## Methods

### Research design

We used a qualitative case study design, using a combination of individual interviews, Focus Group Discussions (FGDs) and observations of the living conditions of the patients. This method allowed for the integration of observations and more consideration of the holistic and unique context of each group and triangulation of findings from these different participants [18]. A group of patients were followed-up after discharge from the hospital where they were diagnosed with TB or DR-TB. We conducted additional interviews with HCWs and family members of patients regarding the services or assistance they provided to patients on TB treatment.

### Study setting and recruitment

We purposefully recruited patients diagnosed with TB who were initially treated for TB in two hospitals and were referred to five clinics (Clinic1-5) in the Western Cape. These 5 clinics are located in informal settlements in the Western Cape. Participants were recruited if they were adult (18 years or older) and had been recently diagnosed with TB or DR-TB and co-infected with HIV. Recruited HCWs were those who had knowledge of the discharge and referral system. All eligible patients were approached during their hospitalisation if they met the study criteria and enrolled if they agreed to be followed up during the course of their treatment. Fourteen of 17 patients agreed to be followed-up. We could only locate 10 of the patients because they gave us accurate addresses. We recruited HCWs after the patients had been referred to specific clinics. The details of the HCWs were secured through the clinic managers of the referral clinics. All HCWs approached agreed to participate in the study. HCWs were approached either face-to-face or through emails. All participants were given information about the study and signed consent forms.

### Data Collection

We conducted one-on-one interviews using semi-structured interview guides to collect data from patients. Each patient was interviewed twice (except one patient who was only interviewed once because she passed away before the completion of her treatment). Each patient was interviewed one to two months after discharge from hospital and again at the completion of treatment, which is about after 20 weeks of treatment based on the Directly Observed Therapy-Short Course (DOTS) plan in South Africa. The two DR-TB patients’ treatment was ongoing, but we secured information of treatment completion of these patients from relevant HCWs at the patients’ referral clinics.

The interview guide for patients included exploring the perceptions and experiences when attending and collecting their TB medication, the support they received at the clinics by HCWs that made it easier or difficult for them to take the TB medication as prescribed, their knowledge of the link in terms of communication between the hospital and clinics about their continuation of TB care at their referral clinics, the patient’s living conditions and the support they received at home. Each interview lasted 45 min to an hour. We audiotaped each interview after securing permission from each participant. We took field notes to complement the audiotaped interviews. The first author, with a wide experience in qualitative research, led the patient interviews assisted by a research assistant who was fluent in the local languages of the patients. This assistant had received training in qualitative research and has contributed to many research activities in the Division of Social and Behavioural Sciences. Although all patients could communicate well in English, the patients were encouraged to speak in their first language. We wrote all the interview questions in English. The research assistant translated and explained them in the local languages of the participants. We provided the translation of the questions, but they were not used as all patients communicated well in English.

The research team also used semi-structured one-on-one interviews with HCWs and family members of patients. Interview guide for HCWs included their experiences working with patients who have been referred to the clinics for continuity of TB care, their knowledge of the linkage to treatment of patients between the hospital and the clinic, and their perceptions of the reasons patients attend or do not attend the clinics that they have been referred to for continued treatment. We interviewed HCWs in quiet spaces, in their offices or in minor halls or canteens in the clinics. Interview guides for the family members included questions regarding the support they rendered to their family patient. We conducted interviews with patients and families in the living rooms of their homes. Family members were interviewed at separate times from the patients.

Focus group discussions were held with some HCWs who were available to meet as a group because of the homogeneity of some of the work in the clinic. The discussions allowed for the exploration of their perceptions as they also provided deeper meaning and/or clarification on some matters [18, 19]. The interview guides had a similar structure and list of questions, but the manner of conducting the discussion was different because it was a group interaction. The questions included experiences working with patients who have been referred to the clinics for continuity of TB care, their knowledge of the linkage to treatment of patients between the hospital and the clinic, and their perceptions of the reasons patients attend or do not attend the clinics that they have been referred to for continued treatment. The interviews were done in the canteens during agreed times that did not interfere with health care provision.

Observations were done throughout the interaction of the participants. In some instances, we were present in clinic meetings and observed the living conditions of the patients and their families. This was to understand some experiences of patients and or HCWs that could not be easily captured through one-on-one interviews. This data collection technique was not an ethnographic “participant observation” that involves prolonged involvement and participation of the researchers. However, important activities and processes regarding TB treatment and referrals that were of importance were documented in field notes that clarified some of the themes from the interviews.

The final sample of participants that provided data saturation was 31. These were 10 patients who had been diagnosed with TB at the hospitals and consented to the followed up to the clinics and homes, 15 HCWs (seven nurses, four TB counsellors, two doctors and two social workers) and six family members. See Table 1 for breakdown of participants, kinds of interviews conducted and number of interviewers during the data collection process.

**Table 1:** Data collection process

Participant category	Number of participants	Type of data collection	Number of interviews
Patients	10	Individual interviews	19
HCWs	15		
Nurses	4	FGDs	1
	3	FGDs	1
Doctors	2	Individual interviews	2
Social workers	2	Individual interviews	2
TB counsellors	4	Individual interviews	4
Families	6	Individual interviews	6
Total	31		35

### Trustworthiness

The researchers maintained reflexivity during the different phases of data collection. This was done by explaining potential biases in the information sheet and during the data collection processes. None of the authors worked in a clinical/TB environment but had knowledge of some of the challenges of continuity of TB care from the literature. We encouraged participants to express their perceptions based on their lived experiences. We avoided any leading questions that would steer participants towards the preconceived ideas and concepts of the authors regarding continuity of care. Documenting our observations as we conducted the interviews also helped in the triangulation of the findings in the different data collecting techniques. The information given to participants included an explanation about the purpose of the research, including the potential benefits to the researchers, the patients, and the wider community. We explained that the research processes would not affect any healthcare provision and/or interfere with family commitments. Due to the study’s potential to inform better health practices and outcomes, we encouraged all participants to be honest in their responses. We used the 32-item COREQ checklist to report key activities including the study design, analysis, and findings (Additional file 1: SI.COREQ checklist) [20]. There was no external peer reviewing of the findings but the analytical process was verified by the second author in order to maintain consistency and explored the potential transferability of study findings by documenting the processes of data collection and analysis.

### Data analysis

We used Miles and Huberman’s qualitative data analysis framework [19]. This approach incorporates the interactive nature of qualitative research and facilitates the identification of patterns and themes. We undertook data reduction, which is a process of reducing the bulk of data to the conceptual focus of the research. The next step was data display, which is a process of highlighting these concepts through pieces of data excerpts, and drawing and finally, verifying conclusions, which is done through an evaluation of how these concepts are linked to the data extracts that produced the key findings of the study. We did the initial coding of the data, including comparing interview themes with field notes, which led to data reduction with verifications of these codes via discussions in the team. We later agreed on excerpts of the data linked to the codes as evidence for each theme and sub-theme generated. We reorganised these themes during the verification process. We used the QSR International NVivo11 software package [21] to facilitate the analytical process and organisation of the data, and also to prevent data loss.

## Results

### Patient demographics and clinic attendance

The mean age of the 10 patients that participated in the study was 33 years. Eight were female. Two participants were diagnosed with MDR-TB. All participants were co-infected with HIV. Only two patients had finished secondary school and one had attained a tertiary education. Nine spoke IsiXhosa as their first language and one spoke Afrikaans. See Table 2 for further details of patients demographics.

**Table 2:** Patient Demographics

Patients Pseudonym	Sex	Age	Highest education completed	First Language	Marital Status	Children	Employed
Aphiwe	M	26	Grade 10	IsiXhosa	Single	No	No
Babalwa	F	29	Grade 11	IsiXhosa	Single	Yes (2)	Yes (Photographer)
Bianca	F	28	Grade 7	Afrikaans	Single	Yes (2)	No
Buhle	F	29	Grade 12	IsiXhosa	Married	No	No
Fezeka	F	49	Grade 7	IsiXhosa	Single	Yes (3)	Yes (Domestic Worker)
Nandipha	F	30	Grade 12	IsiXhosa	Single	Yes (1)	Yes (Baker)
Ndiliswa	F	43	Grade 11	IsiXhosa	Married	Yes (4)	No
Thandiwe	F	42	Grade 11	IsiXhosa	Single	Yes (1)	No
Themba	M	29	Grade 11	IsiXhosa	Single	Yes (2)	No
Zintle	F	29	Tertiary	IsiXhosa	Single	Yes (1)	No

The themes and sub-themes are discussed under three main categories – patients’ socio-economic circumstances, health system challenges, and patients’ negative emotions. The themes under patients’ socio-economic circumstances include family relationships and support, inadequate income and the quest for a disability grant. The themes under health system challenges include inadequate linkage between the hospital and the clinic and patients’ negative emotions of being scared, confused, and embarrassed when accessing care or interacting with HCWs at the clinics. The categories, themes and sub-themes are summarised in Table 3. Also, see Table 4 for patients and HCWs’ description of clinic attendance/ non-attendance and completion of treatment.

Of the 10 patients with whom we conducted home interviews, nine attended the clinic at the expected time, which was after one week of TB pills given to them were finished and they would have attended the clinic for continuity of care. One patient attended the clinic three days after her TB pills were finished because she was feeling sick. Four of the patients stopped attending their clinics during the course of four to 24 weeks of treatment. Five of the patients attended consistently, but one passed away before completion of treatment. The patients, their families and HCWs provided reasons for patients’ attendance and non-attendance at clinics during the course of their treatment. The first set of interviews captured patients’ initial contact with the clinic and how they were responding to treatment and planning for further treatment plans. Most of the significant themes emerged during the second interview with patients. This is between the period of 4–20 weeks of treatment. See Table 4.

**Table 3:** Categories, themes, and sub-themes

Categories	Themes	Sub-themes
Patients’ socio-economic circumstances	Family relationships, support and personal motivation	
	Inadequate income and request for disability grant	
	Patients’ agency among structural problems	
Health system challenges	Inadequate linkage between the clinic and the hospital	Inadequate communication between the hospitals and the clinics
		Inconsistent provision of medication before discharge and inadequate TB education at clinics
Negative emotions	Scared and confused when accessing care	
	Feeling embarrassed at clinics	

**Table 4:** Patients and HCWs’ description of clinic attendance/non-attendance and completion of treatment

Patient’s Pseudonym	Hospital referred from	Referral clinic and location	Attendance at clinic after discharge^a^	Stopped attendance (between 4 – 20 weeks)	Attended till completion of treatment	Completed treatment
Aphiwe	Hospital 1	Clinic 1 & Clinic 4 Khayelitsha	Yes	Yes	No	No
Babalwa	Hospital 2	Clinic 3 –Khayelitsha	Yes	No	Yes	Yes
Bianca	Hospital 1	Clinic 6^b^	No	N/A	N/A	N/A
Buhle	Hospital 1	Clinic 5 –Khayelitsha	Yes	No	Yes	Yes
Fezeka	Hospital 1	Clinic 2-Khayelitsha	Yes	Yes	No	No
Nandipha	Hospital 1	Clinic 1 – Delft	Yes	No	Yes	Yes
Ndiliswa	Hospital 1	Clinic 2 & Clinic 3–Khayelitsha	Yes	Yes	No	No
Thandiwe	Hospital 2	Clinic 2 –Khayelitsha	Yes	Yes	No	No
Themba	Hospital 2	Clinic 3 –Khayelitsha	Yes	No	Yes	Yes
Zintle	Hospital 2	Clinic 5 –Khayelitsha	Yes	No	Yes	Yes

^a^Patients are given 7 days TB pills, which they are expected to take at home after discharge. They are expected to report at the clinic before the pills they received from the hospital are finished

^b^Bianca passed away before attending the clinic. Therefore, we did not interview healthcare workers at this clinic

### Patients’ socio-economic circumstances

#### Family relationships, support and personal motivation

*Cheryl: It was only on Friday when they* [Bianca’s aunt] *came … I don’t have contact with my daughter. She is not part of my life anymore because they are grown-ups, they are adults. It is just now when she came.* [Cheryl explained this in an unpleasant tone]. *Friday was when she came here. Now you understand my inconvenience?*

Apart from the fact that Cheryl had not had a good relationship with Bianca, her present living conditions escalated the problem. We probed this issue further.

*Idriss: You mentioned certain things over the phone – could you clarify some of them for me? Firstly, do you think this* [her home] *is a good place for Bianca to be treated?*

*Cheryl: No.*

*Idriss: Did you try and speak to a family member?*

*Cheryl: Because, number one, it is a bachelor flat. It is only supposed to be for one or two people, but now she is here it makes it very …* [could not find the words]. *I have to keep her here and look after her and I am at work…So that is the inconvenience, and I know with TB you have to have a lot of breathing space. You can see here; it is not much. And at night I cannot sleep with my windows like this. So all in all, it is very inconveniencing. And my personal life, it is being affected….So that is putting a bit of strain on my personal life also.*

Cheryl was apparently not pleased about her daughter being sent to her flat for further treatment. We also observed that Bianca was very frail and weak and needed focused care. Unfortunately, after one month of treatment at home before she could start attending her referral clinic, Bianca had passed away. In spite of the estranged relationship Cheryl had with Bianca, she was not pleased with the manner in which the hospital handled the discharge process.

Another patient, Thandiwe, experienced domestic abuse which hindered her continued attendance at the clinics during the course of her treatment. When she was asked how she was coping with the treatment after discharge from the hospital, she replied:

*Thandiwe: Hey Idriss, I don’t want to cry* [she started crying anyway]. *I was too stressed I guess, because my uncle who was living here came inside here at home and he was the one who was abusing me and everything from an early age. He was the one who made me sick. He is my grandmother’s last-born son. And when he is here I am not motivated to do anything. You know I am sorry to say this, but I hate him so much because I won’t be in this position if it wasn’t for him, but now that he’s left I am just trying to scram my life back together again…I have not been feeling well lately. I have not been going to the clinic either.*

*Idriss: Did you try and speak to a family member?*

*Thandiwe: My grandma knows everything, but you know how old people are. She said, ‘Why didn’t you say anything sooner?’ Because I had to break it to her that I was taking ARVs also … because all these years I have been telling my grandma that I was taking medication for low blood sugar* [Thandiwe was referring to her ARV treatment].

Family relationships were complex and involved expectations that were not always forthcoming. In the second interview with Thandiwe after discharge, the issue of sexual abuse by a family member was brought to the foreground that affected her attendance at the clinic for continued TB treatment. The health system has a structure, which made use of social workers in the hospital and clinics to include family’s participation in their provision of care to the patients. However, with uncooperative relatives, this was often futile.

Conversely, some of the patients expressed having good family support that enhanced their continuity of care. It was also noted that the patients who consistently attended a clinic had better socio-economic circumstances, such as having better financial income through employment or through support from family. The theme of family support was expressed alongside patients’ internal motivation to get better.

Buhle, resided with her husband, brother-in law and other relatives. She received support from them and her sisters who resided elsewhere in better housing locations. Buhle claimed that her family members gave her all the support she needed.

*Buhle: Yes, a lot of support.*

*Idriss: In what ways?*

*Buhle: Most of the time my sister comes. As I tell you, my mother is very sick. They didn’t come. They came with everything that I want; food they gave me if I want, money, if I want. They gave me food at times at work, also my husband supports me, but this week he is on night* [night shift].

*Idriss: Does this help motivate you to go the clinic and take your medication?*

*Buhle: Yes, but again I see many things that happened to people not taking their drugs. And then I told myself, if I take my drugs, I will be better. That is my main motivation.*

Buhle’s last statement focused on actually adhering to treatment. She believed in addition to family support, a positive attitude that came from oneself should be enough for you to attend the clinic and took your treatment consistently. The family members of Buhle as well as Themba confirmed that they supported her in the best way possible and they were happy about the progress that they were making in the treatment of TB. Buhle’s mom, Thandi said:

*Thandi: We all give her hope that she will get healthy again and to encourage her to take the medication.*

Themba’s mom, Saki, also indicated that she would know if he was not taking his medication and if he was not, she would sternly tell him to do so. Saki said:

*Saki: I will ask whether he has taken his medication and he will start scratching his head. And he would say, ‘No I didn’t take it’. And I will make sure that he wakes up and go and take his medication”.*

It is clear that a few of the patients were satisfied with the support that they received from their families. The family members also expressed how they provided this support. However, the patients also mentioned that irrespective of the support, being personally motivated was a key driver for effective continuity of care.

#### Inadequate income and request for disability grant

Patients’ basic living conditions appeared to be barriers to continuity of care. The patients’ demographics in Table 1 shows that seven out of 10 of the participants were unemployed at the time of the study. This finding was triangulated with observations of the living conditions of the patients. The patients were always available at home because of lack of employment. In addition, most of them lived in precarious homes called ‘shacks’. Some of these homes were made of discarded items or scrap materials such as timber and metal sheets, which could be acquired at small cost. Some of the houses barely had a proper chair to sit in. Most of the houses were not only overcrowded, but also lacked running water, better sanitary systems or flushing toilets inside the house and heating facilities to keep residents warm.

Funeka a professional nurse (PN), at Clinic 3, explained that because the patients did not have food, they feared losing disability grants. Funeka explained that some patients wanted disability grants because they needed food, but if that was not forthcoming, they would go to work and would not attend the clinic.

*Funeka: Most of our clients are wanting disability grant because they don’t have food in the house or they were working somewhere else. They cannot come to the clinic because they need to look for work and see how much money they can get for the day to buy food or they work somewhere else where they can’t come back immediately, like on a farm or something like that.*

The social workers and TB counsellors  agreed with the responses made by the nurses. Akhona, a PN who worked at Clinic 2, said that for accessing disability grants, patients must have an identification document (ID), but some of them do not have an ID. The ID card enabled one to access basic social services. As explained above, one of the requirements to access these social services was that one must be able to prove that you were either a South African citizen or permanent resident.

*Akhona: For the others, the problem is an ID issue. Some will be committed to their treatment but the problem is the ID. And they felt that they would stop taking medication because they don’t have food. But if they had the IDs they would benefit to apply for grants.*

From the nurses’ perspectives, the lack of income and quest for disability grant is a driver for continuity of care. Not having an ID to access this grant also compounds the situation for some of the patients. Though the patients did not mention that they were affected by these problems, the nurses may have interpreted this based on what they heard and seen from the attitude of the patients and how these affected their attendance at the clinic. These differences and similarities regarding patients’ continuity of care based on their socio-economic circumstances are briefly discussed under the next theme.

#### Patients’ agency among structural problems

The responses by HCWs about the key factors that influence attendance at clinics included patients’ lack of income and the quest for a disability grant but also mentioned patients’ personal motivation and drug abuse that affected their attendance at clinics. The personal motivation to get better was similar to what some of the patients highlighted themselves.

Most of the clinic nurses indicated that family support and the patients’ willingness to get well are the driving forces to attend clinics for continuity of care. Cebisa, a PN at Clinic 4, mentioned that wanting to get well in order to look after their family is an important motivation.

*Cebisa: I think everything starts from each and every individual because you know what you want to achieve in life. You know you have got goals because those who are coming, they will say ‘I have to come because I have small babies at home. Then I must look after my children so I cannot manage to be sick or die while there is help’. And then the support they are getting from home.*

On the other hand, according to the nurses, lack of personal motivation and drug and alcohol usage play a role when affected patients stopped attending the clinic as well as not adhering to the treatment. The patients in this study, apart from Themba, who said he had a brief drug problem, did not mention having such problems. Khanyi (PN), who worked at Clinic 4, commented on this.

*Khanyi: Sometimes alcoholics, kids on “tik”, we will keep it DOTS and we will try on a weekly basis, it depends. It is the unreliable patients we don’t get on that monthly system, but we try to get working patients on it as quickly as possible, and any patient that we think is reliable will go on to monthly treatment.*

One should note here also from Khanyi’s statement that some patients were given one month’s TB pills if they had responded well to treatment, and were deemed ‘reliable’. This will be explained in detail under health system challenges.

So far, we have discussed some of the socio-economic drivers of continuity of care based on the patients and HCWs’ perspectives, and also highlighted some of the similar and different views of the patients and HCWs. Even though the patients did not mention that lack of income and the quest for a disability grant were affecting their continuity of care, the nurses juxtaposed these as possible barriers as well as the patients’ (mis)use of drugs hindering their continuity of care. They also believed that the choice to get better reflected a strong sense of agency—that some patients had and others did not—that was critical in supporting their attendance at clinic to complete their treatment.

### Health system challenges

Patients’ socio-economic circumstances were not the only factors affecting continuity of care in PHC. Both patients and HCWs have identified several factors that were linked to health system challenges. These health system challenges were the insufficient linkages between the hospital and clinic regarding proper communication and/or ensuring adequate care after discharge from hospital and the lack of patient-centred care by some of the staff at the PHC clinic.

#### Inadequate linkage between the clinic and hospital

The HCWs emphasised that the main link between hospitals and the primary health care clinics was the discharge letter that they gave to the patients. Even though the discharge letter played a significant role by linking the patient from the hospital and clinic, as nearly all the patients attended their clinic, it was a problem to only rely on the discharge letter that were provided to patients because some of discharge letters did not provide adequate information. This partial linkage between the hospital and the clinic are further discussed under sub-themes, which are inadequate communication between hospitals and clinics, inconsistent provision of medication before discharge and lack of adequate TB education/counselling at clinics.

##### Inadequate communication between the hospitals and the clinics

Many HCWs explained that the discharge letter was the main or the only manner of communication between the hospital and clinic. Thami a PN at Clinic 1 explained what happened with the letter once a patient came into the clinic:

*Thami: He’s* [the patient] *got his letter, we basically see whatever is written on the letter and we just take it from there. If he’s started on TB treatment we just continue with all the detail. We open the TB folder, we get all his contact details, we just continue. If he needs to start, he will start with us. The letters are not always very clear. Sometimes you don’t know when the patient really started on TB treatment…there is no telephone number or contact number that we can contact this person or address is not even on it, or it is not clear, it is not the right address.*

According to Thami, getting the letter was not the only issue. Sometimes it did not contain the information they needed. A Nurse Sister at Clinic 5, Ntombi  a PN, explained that they referred such cases to the doctor. According to Ntombi, all patients with missing information were sent to the doctor.

*Ntombi: Sometimes the clients start their treatment from different hospitals, but normally for us it is* [Hospital 2]. *Then they come with the letter. They come to the TB room. We do all the observations, and then we send the client to the doctor. So all those clients are being managed by the doctors. It depends, some of them is normal TB like … GeneXpert or smear, but the first day the patient must first go to the doctor*

According to Ntombi’s response, upon receiving the letter and after having done some “observations” they would send the patient to the doctor. There were patients, however, with either missing information or sometimes badly written referrals that were sent to the doctor. Dr Lina a Medical Doctor (MD) at the Clinic 3 reported that most of the time the notes on the referral forms were clear, but there were times when they were not. However, her main concern was not necessarily a few badly written notes on the letter, but rather that some doctors did not have adequate knowledge of some aspects of patients’ diagnosis and that patients diagnosed with pulmonary TB had “no safety net” when leaving the hospital.

*Dr Lina*: *From the doctor’s point of view, the information that we get from Hospital 2 is of a good quality when they are typed. Sometimes in the Emergency room, we get handwritten, very brief letters. I assume that they are very busy, but a lot of those are very inadequate, particularly with the MDR patients, we can often get wrong medications because again, many, many doctors, most doctors don’t know much about MDR-TB and we occasionally get people with just small mistakes, people with minor problems and they have not adjusted the medication …I think it is one of the challenges that most doctors and nurses have. If they haven’t worked in the TB clinic they are fairly clueless … then a patient will default because between us and the hospital, they didn’t really understand they had TB and so they would take whatever treatment they have got in hospital and we would never see them. So there is no safety net in that sense. If the patient doesn’t come himself, there is no safety net.*

This theme of poor communication leads to another related theme of inconsistent medication given upon discharge of the patients from the hospital. Most importantly, it is good to include here that there was emphasis on Dr Lina’s words that “*We can often get wrong medications because again many, many doctors, most doctors don’t know much about MDR-TB”.* It was not entirely clear if this was an issue pertaining to the specific doctors at the referral hospital where Dr Lina was working or it was a general feeling about the knowledge of MDR-TB of doctors in the country. Either way, if doctors struggled to prescribe medication for MDR-TB patients, then this could be a possible explanation for some patients feeling confused about their diagnosis.

##### Inconsistent provision of medication before discharge and inadequate TB education at clinics

Dr Lina explained further that patients sometimes did not report to the clinic on time because of the amount of medication given to them. Some patients were given TB medication that would last for 7 days and others that would last for a month. They would have to report to the clinic after the medication provided finished. Some nurses were affected by this practice, which they thought raised questions about the DOTS system. This is so because patients were to be observed at the clinic when taking their treatment. If a patient received a month’s supply of TB pills, then this defeats the purpose of the DOTS plan.

*Dr Lina: Now the patient is coming more than a one month later and you say, ‘Why didn’t you come straight from the hospital’ and he will say, ‘No, I was given a month’s treatment’, which is not good for somebody who’s just been diagnosed with TB – that is not good.*

The medication provided to some patients were linked to the kind of TB diagnosis and unique case of the patients among other issues. Therefore this needs to be part of the counselling sessions that were designed for patients. However, one of the key problems identified by some HCWs was that of inadequate TB education. Patient education did happen in the clinics, but there were challenges in doing this effectively. Dr Lina believed that it was not feasible to expect TB education (or counselling, to use her words) to happen effectively at the hospitals because of their workload. She claimed there should be some attempts to speak to patients about TB at the hospital but more of that must be done at the clinic.

*Dr Lina*: *I think with the clinics themselves we are doing as much as we can. We are doing three counselling sessions. We are calling in relatives, community health workers are doing home visits, etc. I don’t think you can expect a busy emergency centre* [at the hospital] *to be doing counselling. I just don’t think so, we just need that safety net. We need to be doing that. I agree with them that the major counselling should be done here….*

Kethiwe, a TB counsellor, informed us about the education given to patients, and noted that they were not sure if patients understood the information. Kethiwe could speak the language that the patients spoke. Nonetheless, it was not sufficient to make her be certain that the information was being understood.

*Kethiwe: We do give education. … there are cases that the client come to us, the counsellors, but you can see that this client doesn’t understand what is going on, then you will give education, but you will see the client doesn’t know what is going on.*

Kethiwe’s last sentence informed us that after going through the process of explaining to patients what TB was all about, there was doubt that they did understand what it was, which explained that the education provided might not have been adequate for some of the patients. We observed that part of the problem of inadequate education could be linked to insufficient staff to manage the influx of patients who needed care. In most of the clinics, there was one doctor who normally saw TB patients, one TB counsellor and a few CHWs who were tasked to do home visits to mostly DR-TB patients. In some clinics, a group of HCWs—nurses, doctors, TB counsellors and CHWs—aligned the services rendered to TB patients and they would convene weekly to discuss activities relating to patients with TB. Based on our observations, one of the problems at the clinic was overcrowding mostly early hours of morning (6am to 11am).

### Negative emotions

#### Scared and confused when accessing care

One consequence of this lack of understanding was that some patients felt scared and confused when accessing care. Fezeka lived in Khayelitsha, and was married, with three children explained her feelings when taking the medication after discharge and her anxiety about continuing to attend the clinic. When asked how consistently she attended the clinic and took her medication, she replied:

*Fezeka: Yes, I do. If you do not take it, the TB will come back. I get more support from the hospital….Because always the nurses *[at the clinic]* will give me the tablets and everything and say you must eat now and take the tablets. The tablets caused the death of my daughter.*

Fezeka’s statement regarding getting “more support from the hospital” and that “the tablets caused the death of my daughter” caused us to probe further on the issue.

*Fezeka: Another doctor take me to another clinic by *[Hospital 2]. *And they told me that my liver is damaged. I told my brother. He was so upset, I was also upset. He wanted to go to the doctor* [at Clinic 3] *asking why he gave me the tablet because my liver was damaged. I am scared. I am so scared.*

*Idriss: What did you do after this?*

*Fezeka: I stopped going there *[*at the clinic*]*, and stopped taking the drugs at some point.* [Fezeka did not explain the exact time that she stopped taking the treatment, but it was between four to 20 weeks because we conducted the last set of interviews in the fifth month of her treatment].

*Idriss: Did you stop going to the clinic to receive the drugs at that time you were scared?*

*Fezeka: Yes. I went to that doctor on Monday and asked him, why didn’t you tell me that my liver was damaged? He said, no, it was not me, it was the nurse who gave you the tablets. Why didn’t you tell me?*

Fazeka and other patients described defining moments in their engagements with HCWs in the hospitals as well as the clinics. Generally, they recounted feeling much better after discharge, but an understanding of the information regarding their treatment including side effects and/or different adaptability to the same treatment seemed to be a problem. Fezeka’s aunt and daughter confirmed that she did stop taking her medication at home, which meant she had also stopped attending the clniic. Fezeka’s aunt, Phumi, said:

*Phumi: Sometimes the patient hides the information from the family … she stopped taking the medication. She thinks she is feeling well now and she can stop taking her medication.*

Phumi’s story did not include Fezeka being scared to attend the clinic and continue taking the drugs. It only affirmed that she did stop taking her treatment. Another patient, Aphiwe, a 28-year-old, lived with his mother in Khayelitsha, explained his experiences with the clinic.

Aphiwe’s story highlights a different reason for not attending the clinic and stopping treatment, but similarly important. He was more “confused” about his care than being “scared”, based on the information given to him about his diagnosis. Aphiwe’s facial expression was not pleasant. He was very concerned about his diagnosis and was not satisfied with the knowledge he had received regarding his sickness. When asked how he was feeling, he replied:

*Aphiwe: I have not been feeling well. I stopped taking the treatment*

We were a bit surprised by Aphiwe’s immediate response as that was the first set of questions we asked in the second interview with him. It appeared he wanted to explain that he had tangible reasons for doing so. We probed further as to why he discontinued the treatment.

*Aphiwe: The doctor that I met *[in Hospital 2]* did not tell me, but at *[Clinic 4*) I did ask them, ‘Doctor, which TB are you treating?’ She told me that it was a normal TB. As the time goes after they discharge me there *[Hospital 2]* I came back again for the treatment *[*at Clinic 4*]*, now the story changed they told me that it is a TB that is close to MDR, but it is not MDR. That is where I started to be confused. They are giving me another treatment {at the clinic*]*. They are giving me another one. So, I don’t know … I am confused.*

The cases of Fezeka and Aphiwe being scared and confused respectively were based on the interaction they had with HCWs, both in the hospitals and clinics. They responded to questions on the kinds of information given to them. Aphiwe and Fezeka seemed to have distrusted the health system because of the mixed messages as well as a failure to explain what may have caused a fatal side-effect or about something else that caused the death of Fezeka’s daughter. As a result of these problems, both patients stopped attending the clinic for continued treatment.

#### Feeling embarrassed at clinics

There were also patients’ reports of feeling embarrassed at clinics. Ndiliswa lived with her grandmother, her aunt, and her husband with their two children, and her own two children as well. She slept on the couch with her two children. She recounted how she had moved to another clinic [Clinic 3] at one time during her treatment because the nurses at her first clinic used to shout at her. This behaviour made her very concerned any time she had to go to the clinic. Ndiliswa’s story of stopping her treatment because of being embarrassed by the nurses when she attended the clinic to receive her treatment also highlights the issue of stigma in some of the clinics, which she said had been a recurring problem in the community.

*Idriss: How do you feel when that* [being embarrassed] *happens?*

*Ndisliswa: I didn’t actually go to the clinic as soon as possible, but when I got there, yoh, those nurses were shouting ‘Why don’t you go to that clinic?’ I told them that a lot of people in my area are going to that clinic. You know how stigma can stay with someone and I don’t like people talking about me, saying she is also taking ARVs and … I mean that also makes you lose your concentration on what you are trying to do, but they said, ‘No you cannot come here’, but I said ’What is wrong in asking for assistance in healthcare? I mean, you can go anywhere and get healthcare, why can’t I come here?*

*Idriss: what happened afterwards?*

*Ndiliswa: I had to stop because there is no taxi there. So you have to practically walk and my legs are still not fit enough, even when I walk long distances, I have to rest in-between, but when you go to* [Clinic 3] *it is like five minutes’ walk.’ You catch a taxi. Why don’t you go there?’ they were shouting. So that doctor wrote a letter to another doctor, and then she said she doesn’t have a problem because she has treated me before. And there you just get your tablets … they* [nurses at Clinic 3] *can really break somebody’s spirit, honestly.*

Some of the participants were keen to suggest what they would like to see in the clinic. Thandi, one of the family members of the participants, was no exception. She indicated what they needed to do at the clinics:

*Thandi: If the clinics could have a proper care for the patients, even the patients themselves could have a way of getting help because of the care that he gets from the clinic. And that will make him go frequently to his appointment. The care is the major part at the clinic. They are not doing enough …They shout at patients and they get very impatient at times.*

A lot of the patients and family members had some good things to stay about the doctors in the clinics, but many bad things to say about nurses. These experiences of the patients affected some of them to attend clinics that were close to their homes. With the socio-economic challenges that patients are faced with, it is likely that if these problems are not addressed, more patients discharged from hospital would drop out from the next level of TB care that has been provided for them.

## Discussion

Effective continuity of care for down-referred TB patients, like many other aspects of TB treatment and care can be a complex challenge for both patients and the health system [4–6, 13, 17, 22]. Our study highlighted a number of factors that can make effective continuity of care either much more or much less likely depending on the personal, family, community and health system circumstances an individual finds themselves in. A better understanding of what these factors are, how they operate, and how some patients might manage to overcome them will be a crucial step in the process of improving TB care at both individual and systems levels.

For all of the participants in our study, in one way or another, socio-economic challenges served as a critical barrier to effective continuity of care. The potential impact of socio-economic circumstances on continuity of care is not a new phenomenon in the literature [14, 23–28]. Like patients in these other studies, TB patients in our study lacked adequate income and other forms of financial support and we described how this affected their care across their treatment journey, from difficulty getting transport to health facilities and lack of food security to painful choices between looking for work and attending clinic appointments. The lack of their own income and/or adequate family support may have led some patients to focus their attention, and anxieties, on securing a disability grant (for some, the sole source of financial support they might have). Disability grants play a crucial role in South Africa in supporting the most basic needs of people dealing with serious illnesses like TB or HIV or other health conditions [29–32]. Research has also shown that if a disability grant is lost, it can have a direct impact on patients’ ability to attend clinic appointments [32]. Challenges with disability grants could also be compounded by problems in the health system such as poor education and counselling on their options [13]. Several patients, for example, described significant confusion around the different timelines and eligibility criteria for disability grants for drug-sensitive TB versus MDR-TB and also reported not being sure who in the health system they could ask for help or advice. Here, health system weaknesses and socio-economic challenges intersect and compound the barriers facing TB patients on treatment.

The role of the family was another critical factor in the lives of patients pursuing continuity of care in this study. As other studies have also highlighted [13, 17, 33, 34], families can provide a crucial form of support to patients diagnosed with TB at home. This support can include both financial and material support (providing accommodation, food, transportation to go to the clinic and medical costs) as well as moral and psychological support and more practical support like helping patients in taking their medication or accompanying them to health facilities. The engagement of family members in the lives of TB patients is of course not always positive. Family members could stigmatise patients living with TB and even withhold the kinds of support described above. The presence of domestic violence within some households also served as an important barrier to continuity of care, likely because of the ways in which conflict between family members could compound existing mental health and psychological challenges [33] like low intrinsic motivation and self-efficacy, which in turn made effective continuity of care that much more difficult.

Some patients, despite facing a range of socioeconomic and family-based challenges, nonetheless managed to exercise some significant degree of agency under these trying circumstances and practice forms of health-seeking behaviour that were an important support for continuity of care. Understanding what health-seeking behaviour is and how to promote it are central concerns in health research [35, 36]. Some authors have interpreted individuals’ agency as primarily an expression of an individual’s personality, intention, will, character or specific up-bringing while others have focused on the ways in which local contexts of health literacy and health service delivery or broader social, political and economic structural factors shape and constrain agency [35, 37]. The patients in this study were clearly significantly constrained by these broader structural factors at work in their lives. This didn’t mean, though, that our study participants were helpless in the face of these constraints. Some were able, often with the support of family or community members, to find ways to overcome these obstacles and maintain their TB treatment. Others, who had less support and perhaps suffered more significant psychological effects from these intersecting medical, personal, social and structural challenges, found it more difficult to exercise the agency needed to stay engaged in care.

Finally, weaknesses in the health system were an important driver of poor continuity of care for patients in this study. Poor linkage between the hospitals and clinics in the TB program was reported across several themes in this study, consistent with reports of other studies conducted in South Africa [4–6, 13, 17]. While problems in health service delivery and health systems are well described in the literature on TB continuity of care, we were struck in this study by the ways in which our participants spoke about the psychological effects of these technical faults in the health system. Patients described being scared of staff, being anxious and confused about their diagnosis and treatment, and being confused about the communication and coordination process that was supposed to ensure continuity in their TB treatment as they moved from the hospital to the clinic. While we identified several administrative issues at work here—such as the lack of clear referral pathways, and poor coordination—underpinning these procedural problems is inadequate patient-centred care of the kind that has been identified as necessary but often missing in many research studies in South Africa and elsewhere [4, 7, 38–40]. While poor patient-centred care enables logistical and communication breakdowns in the health system, it also drives other issues like poor practices of patient education and counselling, and poor treatment of patients in general [13, 41]. The effect of poor patient-centred care is not simply an unsure patient who gets lost in the system and doesn’t receive the care they need—it also produces but a scared, confused, sometimes angry, sometimes despondent, but always more vulnerable patient who will find it that much harder the next time to exercise the energy and agency to re-engage with the health system.

### Limitations

This is a descriptive study of the perceptions and experiences of the participants within a particular socio-economic and cultural context and our findings can thus only be transferred to a limited extent. However, the research methodology and context were documented to inform any future efforts to assess the transferability of these findings in other settings. Despite the efforts made by the authors to minimise any undue researcher influence, some participants may struggle to distinguish a researcher from a medical doctor. It is possible that our presence may have caused some participants to alter their behaviour or provide inaccurate information about their experiences. Also, the research team could not explore the reasons for non-completion of treatment by the two patients who were diagnosed with MDR-TB. However, the clinic verified that they did not complete the treatment and informed us about the circumstances linked to when they stopped attending the clinic. Finally, the data were collected between 2015 and 2017, so it is possible there have been some changes in the rollout of services and patients’ experiences in the clinics in the past five years.

## Conclusion

Poor continuity of care for TB patients should in principle be a fixable problem. While there are no quick fixes for economic marginalisation, TB stigma, or dysfunction in families, the process of down-referring TB patients from existing TB hospitals to existing clinical services should be possible. This is especially true in settings like the Western Cape, South Africa where the public sector health services, despite their many well-known faults and challenges [6, 42] still manage to deliver relatively decent care and treatment outcomes for a highly vulnerable population.

What is clear from our study, however, is that unless a TB program has a well-designed and well-managed system in place for ensuring effective continuity of care, the many community and structural level challenges that TB patients face often quickly overwhelm the capacity of some patients to maintain their TB treatment. Remaining effectively connected to TB care can be difficult at the best of times for even well-resourced individuals and well-functioning health systems. For the patients in these studies, the odds are clearly stacked against them. It is the responsibility of the health system to not only provide the best clinical care possible, but to also engage with patients in a more holistic and patient-centred way, one that understands, anticipates, and works actively to mitigate the many challenges its patients face once they leave the hospital grounds (and indeed, the challenges they face while in hospital).

## Supplementary Information

**Additional file 1. **SI Checklist, COREQ (Consolidated criteria for Reporting of Qualitative research).

## Data Availability

The data are uploaded to the University of Cape Town’s data repository and are available upon reasonable request to the administrator of the UCT data repository, Dr Sanjin Muftic, at dls@uct.ac.za.
