# Supplementary material for: A qualitative exploration of continuity of TB care in clinics after discharge from hospitals in Cape Town, South Africa
Source: BMC Health Serv Res. 2022 Dec 7;22:1489. doi: 10.1186/s12913-022-08880-9 (PMC9727991; doi:10.1186/s12913-022-08880-9)
Supplement: Supplementary file 1 — Additional file 1. SI Checklist, COREQ (Consolidated criteria for Reporting of Qualitative research). [file 12913_2022_8880_MOESM1_ESM.pdf]

Consolidated criteria for reporting qualitative studies (COREQ): 32-item checklist

No. Item	Guide questions/description	Reported on Page #
**Domain 1: Research team and reflexivity**		
Personal Characteristics		
1. Inter viewer/facilitator	Which author/s conducted the interview or focus group?	7-8
2. Credentials	What were the researcher's credentials? E.g. PhD, MD	N/A
3. Occupation	What was their occupation at the time of the study?	31
4. Gender	Was the researcher male or female?	N/A
5. Experience and training	What experience or training did the researcher have?	7
Relationship with participants		
6. Relationship established	Was a relationship established prior to study commencement?	6
7. Participant knowledge of the interviewer	What did the participants know about the researcher? e.g. personal goals, reasons for doing the research	6
8. Interviewer characteristics	What characteristics were reported about the inter viewer/facilitator? e.g. Bias, assumptions, reasons and interests in the research topic	9
**Domain 2: study design**		
Theoretical framework		
9. Methodological orientation and Theory	What methodological orientation was stated to underpin the study? e.g. grounded theory, discourse analysis, ethnography, phenomenology, content analysis	5
Participant selection		
10. Sampling	How were participants selected? e.g. purposive, convenience, consecutive, snowball	6
11. Method of approach	How were participants approached? e.g. face-to-face, telephone, mail, email	6
12. Sample size	How many participants were in the study?	6
13. Non-participation	How many people refused to participate or dropped out? Reasons?	6
Setting		
14. Setting of data collection	Where was the data collected? e.g. home, clinic, workplace	6-8
15. Presence of non-participants	Was anyone else present besides the participants and researchers?	6-8
16. Description of sample	What are the important characteristics of the sample? e.g. demographic data, date	10-11
Data collection		
17. Interview guide	Were questions, prompts, guides provided by the authors? Was it pilot tested?	7-8
18. Repeat interviews	Were repeat interviews carried out? If yes, how many?	7-8
19. Audio/visual recording	Did the research use audio or visual recording to collect the data?	7-8
20. Field notes	Were field notes made during and/or after the interview or focus group?	7-8
21. Duration	What was the duration of the interviews or focus group?	7-8
22. Data saturation	Was data saturation discussed?	8

23. Transcripts returned	Were transcripts returned to participants for comment and/or correction?	N/A
**Domain 3: analysis and findings**		
Data analysis		
24. Number of data coders	How many data coders coded the data?	9
25. Description of the coding tree	Did authors provide a description of the coding tree?	9-10
26. Derivation of themes	Were themes identified in advance or derived from the data?	9-10
27. Software	What software, if applicable, was used to manage the data?	10
28. Participant checking	Did participants provide feedback on the findings?	N/A
Reporting		
29. Quotations presented	Were participant quotations presented to illustrate the themes/findings? Was each quotation identified? e.g. participant number	14-28
30. Data and findings consistent	Was there consistency between the data presented and the findings?	14-28
31. Clarity of major themes	Were major themes clearly presented in the findings?	14-28
32. Clarity of minor themes	Is there a description of diverse cases or discussion of minor themes?	14-28

Developed from:

Tong A, Sainsbury P, Craig J. Consolidated criteria for reporting qualitative research (COREQ): a 32-item checklist for interviews and focus groups. *International Journal for Quality in Health Care*. 2007. Volume 19, Number 6: pp. 349 – 357

Once you have completed this checklist, please save a copy and upload it as part of your submission. Please DO NOT include this checklist as part of the main manuscript document. It must be uploaded as a separate file.
